# Hodgkin Lymphoma As the Underlying Cause of a Chronic Cough

**DOI:** 10.7759/cureus.74778

**Published:** 2024-11-29

**Authors:** Teresa Cardoso, Lúcia Gonçalves

**Affiliations:** 1 Family Medicine, UCSP Alverca do Ribatejo, Unidade Local de Saúde Estuário do Tejo, Lisbon, PRT; 2 Family Medicine, UCSP Paço de Arcos, Unidade Local de Saúde Lisboa Ocidental, Lisbon, PRT

**Keywords:** case report, cough, family medicine, hodgkin, lymphadenopathy, lymphoma, red flags

## Abstract

Hodgkin lymphoma (HL) is a monoclonal lymphoid neoplasm derived from B cells and is one of the most common lymphomas among young adults in developed countries. It typically presents insidiously, often as a painless cervical lymphadenopathy or an asymptomatic mediastinal mass. B symptoms (fever, night sweats, and weight loss), fatigue, pruritus, or alcohol-induced pain may be present along with respiratory symptoms in cases of mediastinal involvement. A definitive diagnosis requires lymph node biopsy for histological and immunophenotypic evaluation.

We report the case of a 24-year-old female smoker with a childhood history of asthma, who presented with a three-month history of dry cough, fever, night sweats, anorexia, and significant weight loss. Subsequently, she developed pelvic girdle pain, pleuritic chest pain, and dyspnea while lying down. A computed tomography (CT) of the chest showed mediastinal lymphadenopathy and consolidation in the right middle lobe with cavitations. Following referral to hematology, a CT-guided mediastinal biopsy confirmed the diagnosis of HL, and a positron emission tomography revealed advanced disease stage IV.

## Introduction

Hodgkin lymphoma (HL) is a lymphoid malignancy characterized by the presence of neoplastic cells (Reed-Sternberg cells) within an inflammatory background [1-7]. In developed countries, it accounts for approximately 10% of all lymphomas [1,2]. According to the World Health Organization classification, HL is divided into two main categories: the classical variant, which is predominant and further subdivided into four subtypes (nodular sclerosis, mixed cellularity, lymphocyte-rich, and lymphocyte-depleted), and the nodular lymphocyte-predominant variant [1,2,4-6,8]. The distribution of HL subtypes varies with geography and the level of economic development [1,3]. Among the subtypes, nodular sclerosis Hodgkin lymphoma (NSHL) is the most prevalent (70%) in economically developed regions [1,2,4,6]. There is a bimodal age distribution, with the majority of histologic subtypes showing one peak in young adults and a second peak in older adults; overall, there is a slight male predominance [1-4,6,7].

The pathophysiology of the disease is not fully established and various associated risk factors are described, including immunosuppression, autoimmune diseases, Epstein-Barr virus infection, family history, and genetic, socioeconomic, and environmental factors [1-3].

Typically, HL evolves slowly and can take weeks to months, indicating a chronic evolution [3,4]. Patients with the classical variant often present with a nontender cervical lymphadenopathy or an asymptomatic mediastinal mass detected on an incidental chest radiography [2-4,6]. The neck is the most common site of involvement [2-4,5]. B symptoms, defined as fever (more than 38°C), drenching night sweats, and involuntary weight loss of more than 10% of body weight over the past six months, occur in less than 20% of patients with early-stage disease, but are present in nearly half of the patients with advanced disease, representing a poor prognosis [2-7]. In cases of extensive mediastinal involvement, symptoms such as dyspnea, cough, or pleuritic chest pain may arise [2-5]. Fatigue is also a common feature, while pruritus is observed in 10 to 15% of cases. Less common presentations include alcohol-induced pain at sites of disease, bone marrow or liver involvement, skin lesions, various laboratory abnormalities, and other paraneoplastic syndromes [2-7,9].

To make a definite diagnosis of HL, an adequate lymph node biopsy is required to determine the histologic subtype and immunophenotyping [2-7,9]. Fine-needle aspirate is insufficient to evaluate architecture and establish the histologic classification [3-6,9]. We describe a case of NSHL that highlights the importance of timely recognition of symptoms in primary care.

## Case presentation

A 24-year-old female, active smoker, with a history of childhood asthma, no regular medication, and a documented allergy to amoxicillin, presented in June 2023 with a three-month history of dry cough, fever, and night sweats. These symptoms were accompanied by non-selective anorexia and approximately 20 kg of weight loss during this period. In August, she reported pelvic girdle pain, left scapular pain, and right costal pain, with a more recent onset of dyspnea in the supine position, leading to easy fatigue.

After multiple visits to the emergency department due to persistent cough and pain, she consulted her primary care physician, who prescribed a series of complementary diagnostic tests, including blood tests, chest radiography, echocardiogram, and chest computed tomography (CT). On physical examination, skin and mucous membranes were slightly pale and no skin lesions were noted. Neck examination revealed no jugular venous distension, no nodules were detected on thyroid palpation, and non-tender subcentimeter right supraclavicular and bilateral cervical lymph nodes were palpable. Cardiac auscultation was normal, and pulmonary auscultation revealed coarse vesicular breath sounds in the right upper lobe without adventitious sounds. The abdomen was soft, nontender, with no palpable nodes, and the liver edge was palpable, but without hepatosplenomegaly. Right inguinal lymрhаԁеոoраthy was also detectable.

**Figure 1:**
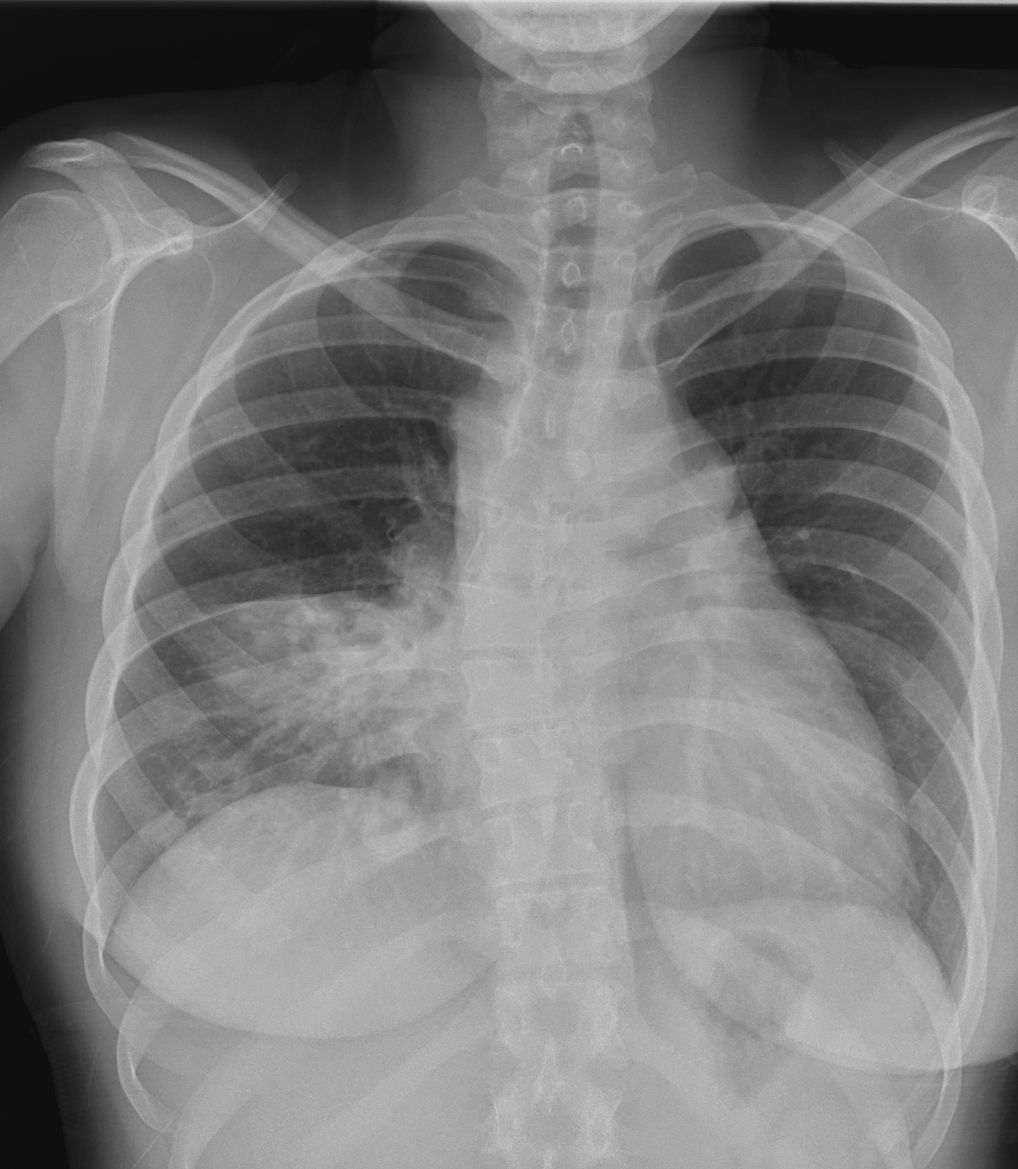
Chest X-ray: anteroposterior erect view Mediastinal widening and consolidation in the right middle lobe, as well as air bronchograms bilaterally.

**Figure 2:**
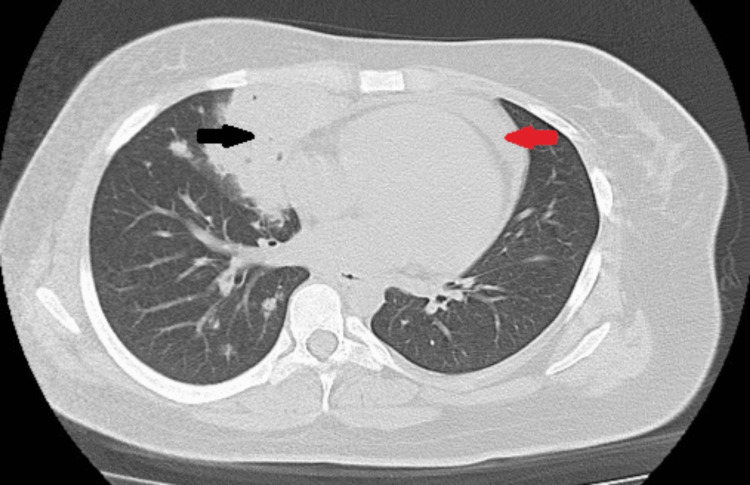
Chest computed tomography: axial plane Mediastinal lymphadenopathies, consolidation in the right middle lobe with cavitary lesions (black arrow), several small centrilobular nodules scattered in the right lung, moderate circumferential pericardial effusion (red arrow).

Laboratory tests revealed new-onset microcytic hypochromic anemia, leukocytosis with neutrophilia, thrombocytosis, and an elevated C-reactive protein and erythrocyte sedimentation rate (Table 1). Renal, hepatic, and thyroid function tests showed no abnormalities. A chest radiography and echocardiogram showed a mass and mediastinal enlargement, and a small pericardial effusion without hemodynamic compromise, respectively (Figure 1). Subsequently, a chest CT highlighted mediastinal lymphadenopathies, the largest in the anterior mediastinum measuring 8 cm x 3.5 cm, mild pericardial effusion, and consolidation in the right middle lobe with cavitary lesions and air bronchogram (Figure 2).

**Table 1:** Laboratory results. Abnormal values are highlighted in bold. Abbreviations: Hb = Hemoglobin; MCV = Mean corpuscular volume; MCH = Mean corpuscular hemoglobin; ALT = Alanine aminotransferase; AST = Aspartate aminotransferase; TSH = Thyroid-stimulating hormone; T4 = Thyroxine.

Parameter	Value	Normal range
Hb	9.2 g/dL	12-16 g/dL
MCV	78 fL	80-98 fL
MCH	24 pg	28-32 pg
Leukocyte count	14x10^3^/µL	4.5-11x10^3^/µL
Absolute neutrophil count	11x10^3^/µL	1.5-7.7x10^3^/µL
Absolute lymphocyte count	1.69x10^3^/µL	1-4x10^3^/µL
Platelet count	501x10^3^/µL	150-450x10^3^/µL
C-reactive protein	25.15 mg/dL	≤0.8 mg/dL
Erythrocyte sedimentation rate	120 mm/h	0-20 mm/h
Serum creatinine	0.65 mg/dL	0.50-1.10 mg/dL
ALT	16 U/L	10-40 U/L
AST	8 U/L	10-40 U/L
TSH	1.361 mU/L	0.5-4 mU/L
Free T4	0.95 ng/dL	0.8-1.8 ng/dL

**Figure 3:**
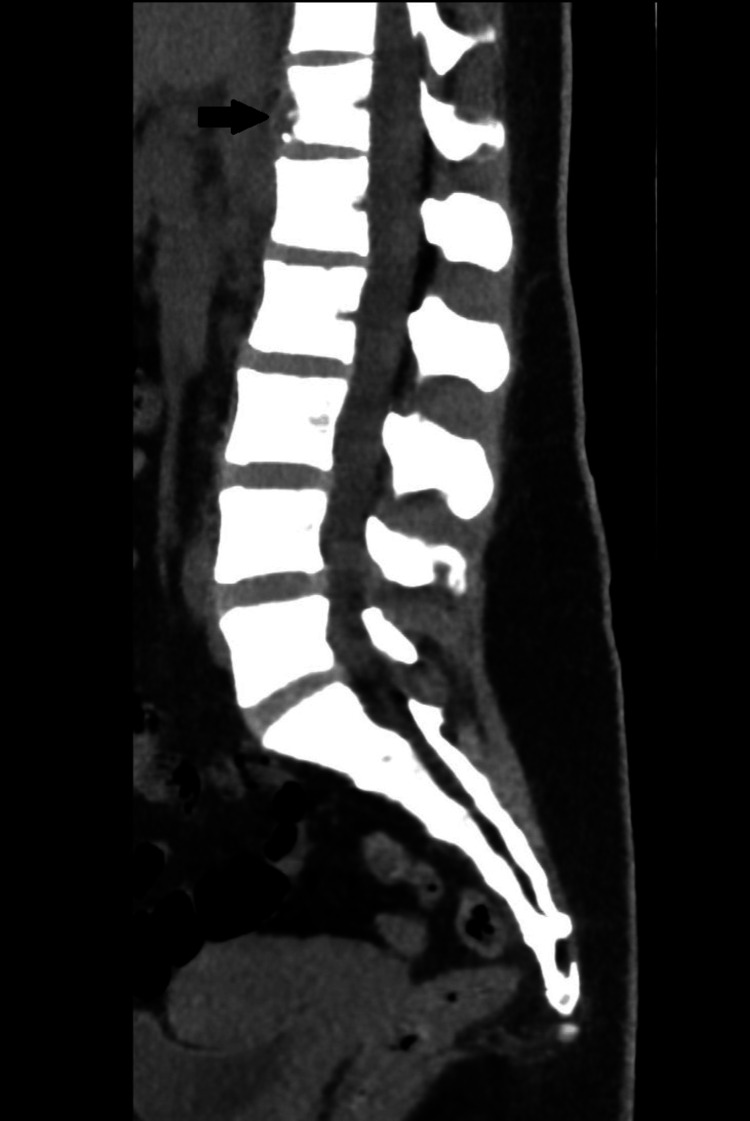
Lumbosacral computed tomography: sagittal plane Radiolucent lesion involving the T12 vertebral body (black arrow).

The patient was referred for assessment by the internal medicine department of the local hospital in September. She received treatment with levofloxacin 500 mg daily for seven days, but without symptomatic improvement. Sputum samples were collected for the detection of *Mycobacterium tuberculosis*, which yielded negative results. A lumbosacral CT scan showed a radiolucent lesion on the anterior aspect of T12 with slight prevertebral extension (Figure 3).

**Figure 4:**
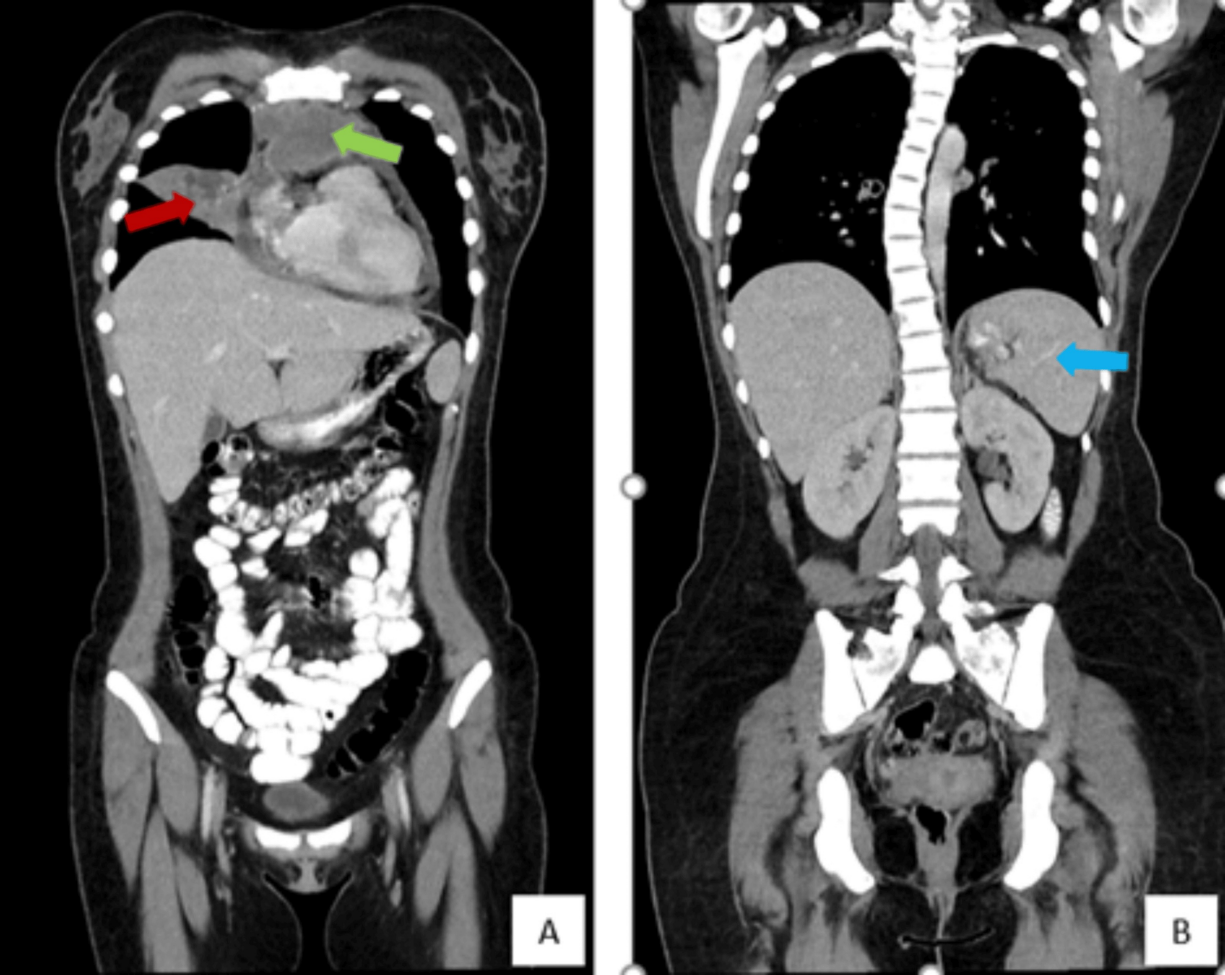
Thoraco-abdominopelvic computed tomography - coronal plane. (A) Lymphadenopathic involvement of the mediastinum, particularly in the thymic region, with a central necrotic component extending over an 8.8 cm area (green arrow); tumoral involvement of the pulmonary parenchyma in the middle lobe with a large area of consolidation and a necrotic center measuring 9 cm at its longest axis (red arrow); (B) Splenomegaly with a maximum axis of 13.7 cm (blue arrow).

**Figure 5:**
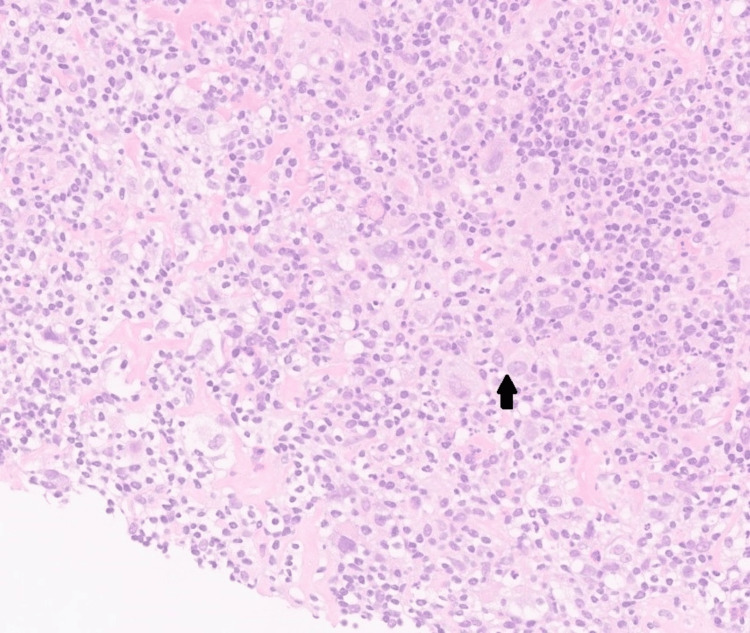
Histopathological images of the mediastinal biopsy. Neoplastic, large bilobed cells with two or more nuclei with eosinophilic nucleoli, consistent with Reed-Sternberg cells, are present (black arrow).

Under these circumstances, the patient was then referred to the hematology department at the Portuguese Institute of Oncology. Further analytical studies were performed, including viral serologies and autoimmune testing, all of which returned negative results. The patient underwent a CT-guided percutaneous core-needle biopsy of the mediastinum that proceeded uneventfully. The thoracic-abdominopelvic CT scan revealed extensive findings, including multiple adenopathies in the mediastinal and pulmonary hilar compartments bilaterally. There was evidence of involvement of the pericardium, pleura, and pulmonary parenchyma in the right middle lobe (Figure 4A). Additionally, lymphadenopathy was noted in the right supraclavicular and axillary regions, as well as in the celiomesenteric region and along the common iliac axis bilaterally. Splenomegaly was also observed, accompanied by several subcentimeter nodules located in the lower third of the spleen (Figure 4B). One week later, the anatomopathological report confirmed the diagnosis of NSHL (Figure 5).

**Figure 6:**
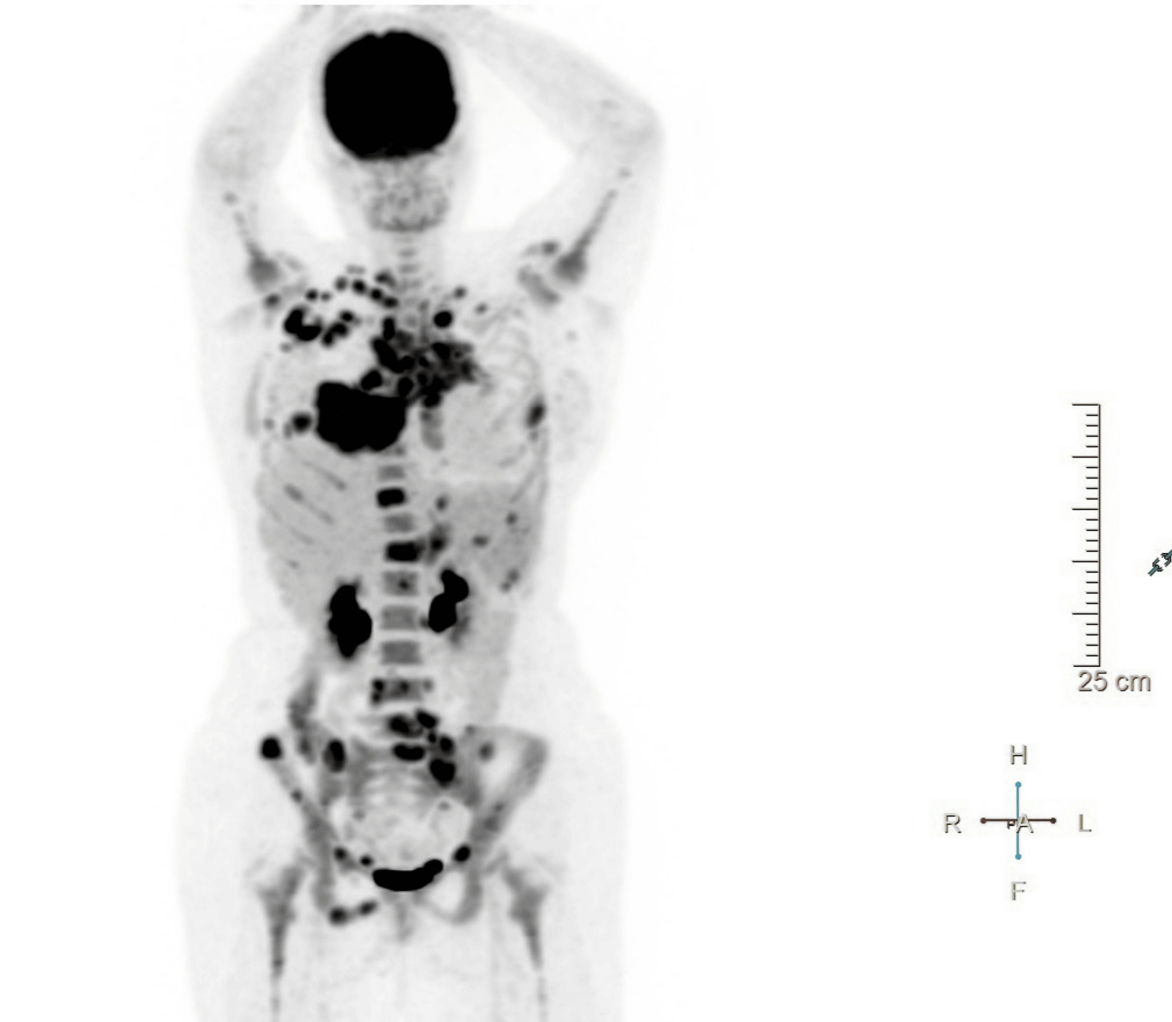
Positron emission tomography: coronal plane Widespread supraclavicular, mediastinal, hilar, abdominal, and pelvic lymphadenopathy in addition to involvement of the lungs, spleen, and bones.

A positron emission tomography (PET) was requested for disease staging, which showed abnormal fluorodeoxyglucose (FDG) uptake at the supradiaphragmatic lymph nodes (with extensive involvement and bulky disease), as well as adenopathies in the infradiaphragmatic region and pulmonary, bone, and splenic involvement (Figure 6). According to the Lugano classification, these findings were consistent with advanced stage IVB HL.

In a multidisciplinary consultation in early October, initial treatment with the gold-standard ABVD chemotherapy regimen (doxorubicin, bleomycin, vinblastine, and dacarbazine) was proposed. Currently, the patient is clinically stable and partially responding to the ongoing treatment. Close monitoring and follow-up, both in primary and secondary healthcare settings, is being provided.

## Discussion

Cough is a highly prevalent symptom in clinical practice and is usually associated with benign conditions [10]. In this patient’s case, nonspecific respiratory symptoms like cough or dyspnea could be attributed to pre-existing asthma. However, the constellation of clinical findings raised other diagnostic concerns.

The diagnosis of lymphoproliferative diseases can be challenging, as numerous other conditions may present similarly with lymphadenopathy accompanied by fever, sweats or weight loss [2,3,5]. Here, in the initial diagnostic workup, an infectious process was considered the primary diagnosis. Given the lung consolidation, the patient was treated with broad-spectrum antibiotics for pneumonia; however, her symptoms showed no improvement. Pulmonary tuberculosis (TB) was subsequently considered a probable diagnosis. The symptomatology, right-sided lung consolidation, and enlarged hilar and mediastinal lymph nodes observed on the chest radiography and CT supported this hypothesis. However, TB tests were negative and the vertebral lesion detected on the lumbosacral CT scan raised suspicion of an alternative etiology. This diagnostic dilemma is well-documented, with multiple reported cases of misdiagnosis where presumed TB masked an underlying HL, to the extent that TB has been called “the great imitator” [11-15]. In these cases, patients often showed no response to anti-TB therapy, while microbiological and molecular tests were negative; further investigation through core-needle or excisional biopsies ultimately revealed the diagnosis of HL [11-15]. Beyond TB, other causes of generalized lymphadenopathy with systemic symptoms, such as systemic lupus erythematosus (SLE), human immunodeficiency virus (HIV) infection, sarcoidosis, and certain medications, should be excluded [2,3,5,16,17]. SLE was also considered unlikely here due to the absence of other major clinical features, such as leukopenia, arthralgias, mucocutaneous lesions, or renal involvement, along with a negative autoimmune panel. Additionally, the patient's viral serologies were negative. It is worth noting that HL incidence is actually elevated both in patients with immunodeficiency and autoimmune diseases [1]. Though sarcoidosis can involve the lungs and thoracic lymph nodes, the imaging findings in this case were inconsistent and the biopsy did not confirm this diagnosis. Certain medications, such as allopurinol or atenolol, may also trigger reactions with fever and lymphadenopathy [16]. However, our patient was not on any chronic medications. While imaging techniques play a pivotal role in the diagnostic process, histopathological evaluation via biopsy is often a decisive step when final diagnostic uncertainty persists, remaining indispensable for establishing a definitive diagnosis of HL [2-7,9,11-16]. HL must also be distinguished from anaplastic lуmрhоma and various other non-HL, a distinction made possible by identifying unique morphological, immunophenotypic, and molecular features on lymph node histology [2,3,5].

In the reported case, staging with PET-FDG revealed widespread disease, including bulky mediastinal mass and extranodal involvement. PET is the mainstay of imaging for staging HL, providing detailed characterization of the disease, extent of progression, and identifying potential organ involvement. Pretreatment evaluation of a patient with HL involves establishing the stage of disease, assessing medical comorbidities, performance status, and overall medical fitness [2-7,9,18-20]. Counseling on fertility preservation should also be addressed [2-6,18-20].

Treatment of advanced (stage III-IV) classic HL typically involves chemotherapy. Consolidative radiation therapy is not routinely employed, as it has not demonstrated a benefit in overall survival for patients who respond to chemotherapy. The ABVD regimen proposed for this patient remains one of the most commonly used first-line treatments for HL, providing favorable long-term survival rates even in advanced stages [2-4,6,7,19,20]. Approximately 70% to 80% of younger patients with stage III-IV HL remain disease-free at the 10-year mark following conventional chemotherapy [2-4].

All healthcare providers should be diligent in the early detection and timely referral of patients with serious conditions. Family medicine physicians, in particular, are often the first point of contact for patients within the healthcare system. This case illustrates the importance of broadening the differential diagnosis when evaluating chronic cough, especially if persistent lymphadenopathy and constitutional symptoms are evident. It also highlights that some infectious, autoimmune, and other inflammatory processes can mimic neoplasms like HL, making it essential to differentiate between these entities and establish a definitive diagnosis. Raising awareness on how to conduct a thorough differential diagnosis could help prevent misdiagnoses or delayed recognition of various conditions. Early identification of warning signs and initiation of timely diagnostic workups can greatly impact patient outcomes by ensuring prompt referral to specialists and swift initiation of treatment.

Moreover, ongoing longitudinal care is equally crucial in the management of oncology patients. As primary care providers who are working closely with secondary care, we play a key role in monitoring for treatment-related side effects, disease recurrence, and overall patient well-being. Regular follow-ups allow for reassessment and the management of any late complications or comorbidities that may arise from both, the disease and its treatment. By fostering a strong, patient-centered approach, family doctors ensure a comprehensive care pathway, supporting not only the acute management of illness but also the patient’s long-term health and quality of life.

## Conclusions

In conclusion, this case emphasizes the importance of maintaining a broad differential diagnosis and remaining vigilant for less common diagnoses when confronted with persistent, non-specific symptoms such as chronic cough and constitutional symptoms. HL, though uncommon, should remain a diagnostic consideration, especially when red flags such as B symptoms are present.

Timely recognition and referral to specialized care can have a significant impact on patient outcomes, especially in more advanced stages, where timely intervention is critical. A multidisciplinary approach is vital to ensure that management is personalized to the patient's needs. In addition, the ongoing involvement of the primary care team is crucial, not only in addressing comorbidities and supporting the patient’s overall health but also in facilitating coordination with specialists to ensure seamless and integrated care throughout the treatment journey.
